# Experimental Parametric Relationships for Chip Geometry in Dry Machining of the Ti6Al4V Alloy

**DOI:** 10.3390/ma11071260

**Published:** 2018-07-23

**Authors:** Yezika Sánchez Hernández, Francisco Javier Trujillo Vilches, Carolina Bermudo Gamboa, Lorenzo Sevilla Hurtado

**Affiliations:** Department of Manufacturing Engineering, EII, Universidad de Málaga, E-29071 Málaga, Spain; yezika.sanchez@uma.es (Y.S.H.); bgamboa@uma.es (C.B.G.); lsevilla@uma.es (L.S.H.)

**Keywords:** Ti6Al4V alloy, chip geometry, dry machining, parametric models

## Abstract

The Ti6Al4V alloy is included in the group of difficult-to-cut materials. Segmented chips are generated for a wide range of cutting parameters. This kind of chip geometry leads to the periodic variation of machining forces, tool vibrations, and work part-tolerance inaccuracies. Therefore, the analysis of chip morphology and geometry becomes a fundamental machinability criterion. However, few studies propose experimental parametric relationships that allow predicting chip-geometry evolution as a function of cutting parameters. In this work, an experimental analysis of the influence of cutting speed and feed rate on various chip-geometric parameters in dry machining of the Ti6Al4V alloy was carried out. In addition, the chip morphology and chip microstructure were studied. A clear dependence of certain chip-geometric parameters on the cutting parameters studied was found. From the experimental data, several parametric relationships were developed. These relationships were able to predict the evolution of different geometric parameters as a function of cutting speed and feed, within the tested range of values. The differences between the proposed models and the experimental data were also highlighted. These parametric equations allowed quantifying the value of parameters in which the trend was clear.

## 1. Introduction

Titanium alloys exhibit exceptional properties that make them a good choice for many industrial applications, such as aeronautic, power-generation, or biomedical industries. Due to their low density, very high strength-to-weight ratio (even higher than wrought aluminum alloys), and excellent corrosion resistance at high temperatures, these alloys are widely used in structural parts of aircrafts and components which work under extreme environmental conditions (turbine blades, combustion chambers, etc.). In addition, their chemical inertness at room temperature is highly appreciated in the manufacture of medical implants [1,2,3].

Depending on their crystalline structure, titanium alloys can be classified into two large groups: (a) corrosion-resistant alloys (α), and (b) structural alloys (close α, α + β, and β alloys). Regarding the aeronautical industry, structural alloys are the most common [4,5]. In particular, the Ti6Al4V (α + β) alloy is within this last group, and represents 60% of the titanium used in this industry [6]. It is mainly used in the construction of components for engines and structures, either alone or hybridized with other materials such as carbon fiber reinforced polymer (CFRP) to form fiber metal laminate (FML) structures (CFRP/Ti) [7].

Machining (mainly turning, milling, and drilling) is one of the most common formation processes used in the manufacture of these structures [8]. Despite the good properties of the Ti6Al4V alloy, it is included in the group of difficult-to-cut materials, due to several problems not yet solved [9]. On one hand, its low thermal conductivity avoids a fast evacuation of the heat generated in the cutting area. As a result, the cutting temperature increases, so tool wear and tool life are negatively affected. On the other hand, it shows high chemical reactivity above 500 °C. So, most tool materials are not suitable for machining this alloy because of their chemical affinity, which results in fast tool wear [10,11]. Furthermore, this alloy exhibits a low elastic modulus, which results in higher workpiece deformation and springback, inducing chattering and dimensional accuracy problems [12]. Finally, when machining this alloy, segmented chips (saw-tooth chips or serrated chips) are generated at relatively low cutting speeds. This chip geometry leads to the periodic variation of machining forces and tool vibrations, which may cause tool fatigue, breakage, and workpiece tolerance inaccuracies [13,14,15].

Traditionally, Ti alloys are cut at low cutting speeds, using cutting fluids in order to reduce the cutting temperature and to prevent tool wear. However, the use of these substances results in environmental pollution and fluid-saving problems. Thereby, new alternative methods to replace cutting fluids were developed [16]. Among them, dry machining minimizes the negative environmental effects, thermal shock in discontinuous machining operations, and health risks for workers [17,18,19]. However, the total absence of cutting fluids causes quick tool wear and the degradation of workpiece surface integrity [20,21]. Under these extreme conditions, the correct selection of cutting parameters (feed, cutting speed, and cutting depth) is of crucial importance to improving the machining performance from different points of view (environmental, economic, energy, and functional) [22].

Within this context, the analysis of chip morphology becomes a fundamental machinability criterion. The chip morphology provides information about the cutting operation’s stability and the material response (thermal, mechanical, etc.) [23,24]. As previously commented, the segmented chip obtained while machining the Ti6Al4V alloy directly affects cutting forces, cutting temperature, tool wear, and workpiece surface quality. In addition, machining this alloy produces a large amount of continuous chips that must be evacuated and handled efficiently. This fact becomes a transcendental problem in the one-shot dry drilling of stacked materials (composite–metal assemblies) [25]. Hence, a correct understanding of cutting conditions, giving rise to an easier-to-handle chip, minimizing tool wear, and improving the surface integrity of machined parts, is highly important [26].

A large number of studies can be found in the literature devoted to the analysis of chip morphology and the chip-formation mechanism of the Ti6Al4V alloy. Nevertheless, this mechanism is not completely understood [27]. There are two main theories which explain the formation of segmented chips in this alloy: (a) thermoplastic deformation, and (b) fracture [8,28]. The first one supports that segmented chips are the result of the formation of adiabatic shear bands within the primary shear zone, caused by the predominance of thermal softening over strain hardening. The second theory explains chip segmentation through crack initiation and propagation from the outer chip surface toward the primary shear zone. Both theories are not mutually exclusive. Some authors suggest that the adiabatic shear band is the precursor of crack initiation [29,30,31,32].

The study of chip geometry provides valuable information about the energy consumption involved during machining, tool wear, and tool life, and the effect of machining on machined parts (surface quality, geometric deviations, residual stress, etc.). Because of this, a significant amount of research focuses on the analysis of several chip-geometric parameters (segment ratio, chip thickness, segment width, shear angle, shrinkage factor, chip segment frequency, etc.) of the Ti6Al4V alloy, and their evolution with cutting parameters [33,34].

Some studies analyzed chip geometry from an analytical point of view. Thus, several theoretical models were formulated [35,36,37]. Notwithstanding, there are many interacting variables involved in machining, and therefore, in the chip generation process. As a result, analytical studies are complex, and they require the application of a set of simplifications which usually lead to inaccurate predictions of various chip-geometric parameters [8,33,34]. Other studies used numerical models (finite element method, FEM) to simulate the chip generation process [8,23,27,38,39,40,41]. These models can be interesting as long as the constitutive laws of the material (tool and workpiece) are well defined. Regrettably, these constitutive laws are often incomplete. As a consequence, these models frequently overestimate or underestimate certain chip-geometric parameters [27,33].

Longitudinal and transverse chip sections can also be experimentally observed using metallographic techniques (hot mounting, polishing, and etching) combined with stereoscopic optical microscopy (SOM) or scanning electron microscopy (SEM). The measurement of chip-geometric parameters can be carried out using a digital-image-capture system and image-processing software. In this regard, a lot of research that analyzes the influence of cutting parameters on chip geometry can be found in the literature [3,10,31]. However, few studies propose experimental parametric models that allow predicting the evolution of geometric parameters as a function of cutting parameters [20,22,42].

In this work, an experimental analysis of the influence of cutting speed and feed rate on various chip-geometric parameters was carried out in dry machining of the Ti6Al4V alloy. In addition, the chip morphology and chip microstructure were studied. From the experimental data, several parametric models were developed. These models were able to predict the evolution of different geometric parameters as a function of cutting speed and feed, within the range of values tested.

## 2. Materials and Methods

Table 1 shows the composition of the tested Ti6Al4V alloy. This composition was obtained using arc atomic emission spectroscopy (AES).

**Figure 1:**
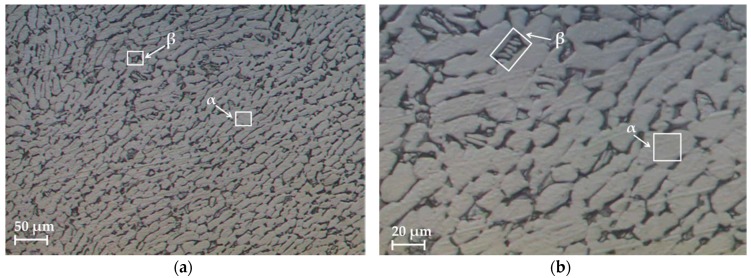
(**a**) Stereoscopic optical microscopy (SOM) images of the microstructure of the tested Ti6Al4V alloy and (**b**) amplification and identification of α and β.

Stereoscopic optical microscopy (SOM) techniques were used to observe the alloy microstructure (Figure 1a). A bimodal structure (α/α + β), which is typical in this alloy, can be observed. The structure was formed by a globular primary α phase (light color in the image) where aluminum was concentrated, and a transformed β phase containing an acicular α phase (lamellar structure, dark color in the image), where vanadium was concentrated (Figure 1b) [43,44]. The samples were polished prior to carrying out SOM.

The experimental study was designed to evaluate the influence of cutting parameters on the chip geometry of the Ti6Al4V alloy. For this purpose, a set of machining tests was carried out. Various combinations of cutting parameters (cutting speed (*v_c_*), feed rate (*f*), and cutting depth (*a_p_*)) were selected. A factor-by-factor study was applied, and their values are shown in Table 2. The range of values was chosen based on industrial requirements. It is necessary to point out that low values of cutting speed were selected to prevent quick tool wear [4,9].

In order to guarantee the repeatability of the tests, 10 specimens were machined for each combination of cutting parameters. Thereby, a total of 120 specimens were tested. All tests were conducted in a parallel lathe, and were performed dry, with the aim of using techniques with a low environmental impact.

**Figure 2:**
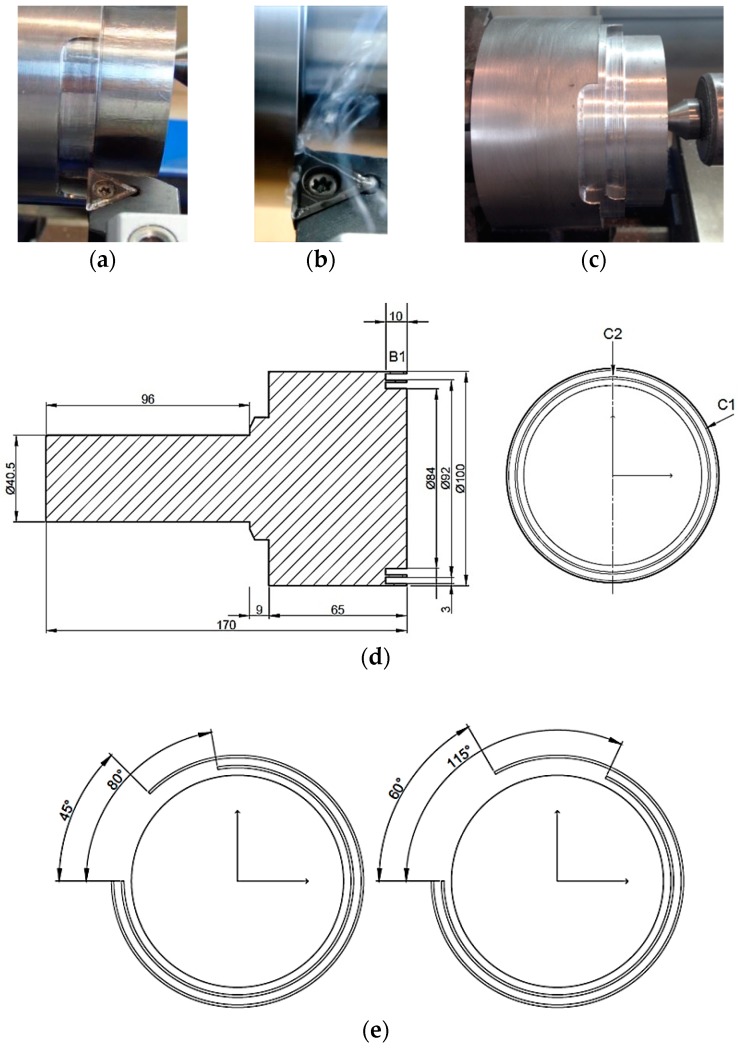
(**a**) Orthogonal cutting disposition; (**b**) initial machining instance; (**c**) sample for several cutting tests; (**d**) test sample design (mm); (**e**) relief zone design.

Turning tests were carried out in an orthogonal configuration to minimize the influence of geometric variables, which could make the process more difficult to characterize (Figure 2) [27].

A combination of the tool insert, TCMT 16T308-F1, and the tool holder, STGCL 2020K16, provided geometrical features very close to an orthogonal configuration (Figure 2a,b). The tool used had WC-Co inserts coated with TiCN/Al_2_O_3_ [2,3]. A new tool was used for each cutting test in order to maintain the same initial conditions.

Specimens were designed with a tailored geometry to maintain orthogonal conditions across the tests, and to achieve different ranges of cutting speed and feed rate. Different grooving operations were carried out on a billet (*L* = 170 mm, *D* = 105 mm) to achieve a tubular geometry (Figure 2c). Specimens were formed by two crowns, corresponding to the two diameters machined previously (C1 and C2, Figure 2d). Each crown was machined with a specific thickness equal to *a_p_*. B1 represents the work section at a 10-mm distance, equivalent to the total cut magnitude that corresponds to a certain combination of cutting parameters. Additionally, a relief zone was established, eliminating a sector of the crowns (Figure 2e), to ensure that the spindle reached a permanent regime. A 96-mm-long cylinder (on the left) was implemented to ensure correct fastening.

The chip generation process was monitored online using a digital camera. The chip samples were collected, photographed, stored, and codified after machining for further observations. Metallographic techniques were used to prepare the chip samples for observation (offline). The chip samples (longitudinal and transverse sections) were embedded in epoxy resin, before being polished and chemically etched (Kroll’s reagent, 50 mL of H_2_O + 2 mL of HF + 5 mL of HNO_3_) to facilitate the observation.

An inverted metallurgical microscope (EPIHOT 280 NIKON, Tokyo, Japan) was used to observe the chip specimens. The SOM images were obtained using a CF Infinity Optical System (1.5× to 400×). Chip measurements were carried out using a digital-image-processing software (Omnimet BUEHLER, Lake Bluff, IL, USA).

**Figure 3:**
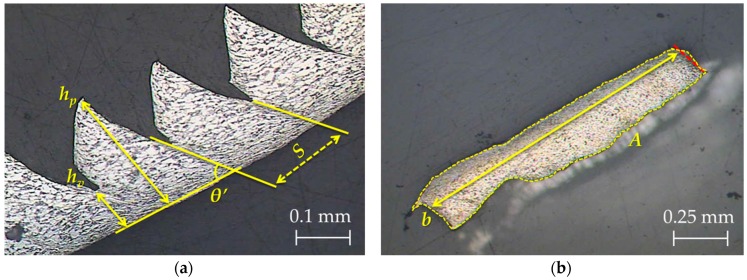
(**a**) Longitudinal and (**b**) transverse chip section measurements.

Figure 3 shows the geometric parameters measured on the longitudinal and transverse chip sections, where *h_p_* is the height of the peaks, *h_v_* is the height of the valleys, *S* is the segment width, *θ’* is the complementary shear angle on the longitudinal section, and *b* and *A* are the chip width and area, respectively, on the transverse section (measured along the adiabatic shear band). To obtain *b*, a perpendicular line was drawn to the farthest point, starting from the base of a line approximately parallel to the chip edge.

The shear angle, *θ*, can be indirectly obtained through its complementary angle, *θ’* (Figure 3), as presented in Equation (1).

(1) $$\theta =\frac{\pi }{2}-\theta^{\prime }$$

Likewise, the shrinkage factor (*ζ*), the segment ratio (*G_s_*), and the equivalent chip thickness (*t_c_*) can be calculated with Equations (2)–(4), respectively, where *γ* is the rake angle on the tool, assuming a constant volume and plane-strain hypothesis [22,33,34].

(2) $$\zeta =\frac{\sin\theta }{\cos\left(\theta -\gamma \right)}$$

(3) $$G_{s}=\frac{h_{p}-h_{v}}{h_{p}}$$

(4) $$t_{c}=h_{v}+\frac{h_{p}-h_{v}}{2}$$

A total of five samples were analyzed, measuring each parameter three times, resulting in a total of 15 measurements for each combination of cutting parameters. The results for the various geometric parameters analyzed were expressed as the average value of these measurements.

## 3. Results and Discussion

### 3.1. Chip Morphology and Microstructure

**Figure 4:**
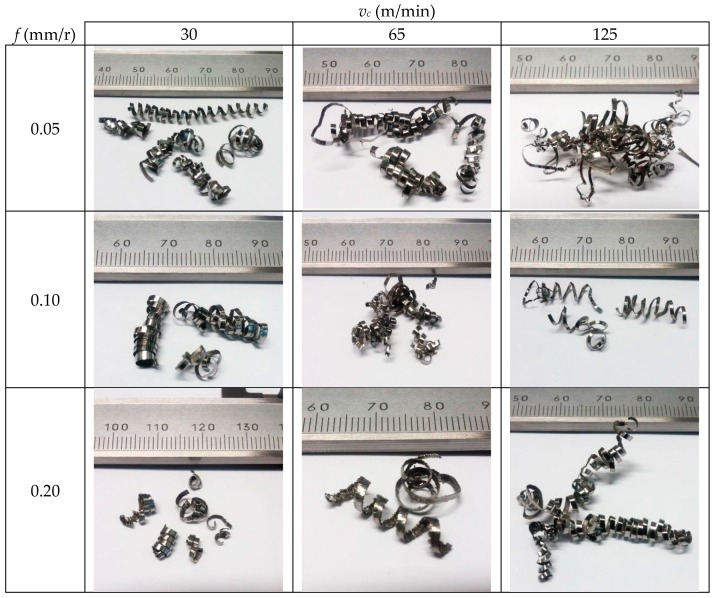

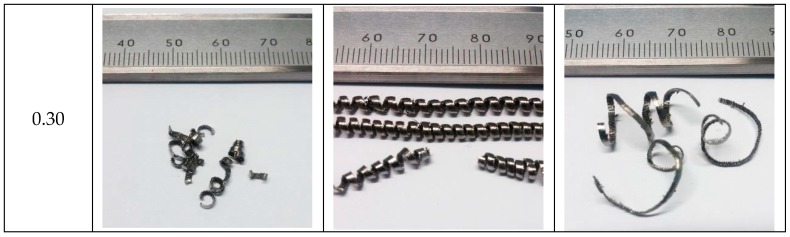
Evolution of the chip morphology as a function of cutting speed (*v_c_*) and feed rate (*f*).

Figure 4 shows the evolution of the chip morphology as a function of the cutting parameters, *v_c_* and *f*. In general, the chip morphology is segmented and remains continuous across a wide range of *v_c_* and *f* studied. Thus, the obtained chip is very difficult to evacuate and handle. This is a consequence, on one hand, of the high plasticity levels of this alloy, which makes it difficult to attain its shear limit. On the other hand, its low thermal conductivity results in thermal softening, which compensates for strain hardening and makes the chip more difficult to break [10,28,30,31].

**Figure 5:**
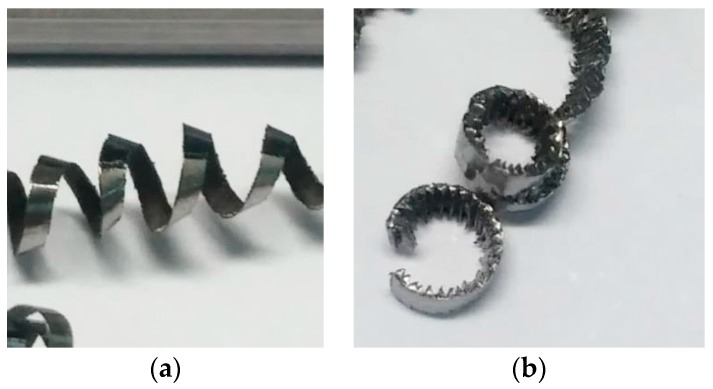
Chip morphology for (**a**) *v_c_* = 30 m/min, *f* = 0.05 mm/r, and (**b**) *v_c_* = 30 m/min, *f* = 0.30 mm/r.

This fact becomes more noticeable when *v_c_* was increased and *f* was reduced. In the lowest feed-rate range (0.05–0.10 mm/r), the chip morphology was continuously helical (Figure 5a) and showed a strong tendency toward forming chip nests for 0.05 mm/r. However, the chips tended to be tubular and more fragmented for the highest feed-rate range (0.20–0.30 mm/r) combined with low cutting speeds (30–65 m/min) (Figure 5b). For these values, the cutting forces were higher and the thermal-softening effects were lower; therefore, chip breaking was easier to achieve. From a machinability point of view based on chip control, the best results came from the highest values of feed rate and cutting speed. However, if other machinability criteria are taken into account, such as tool wear, cutting forces, superficial quality of parts, or temperature (not analyzed in this study), the results may present the opposite trend.

**Figure 6:**
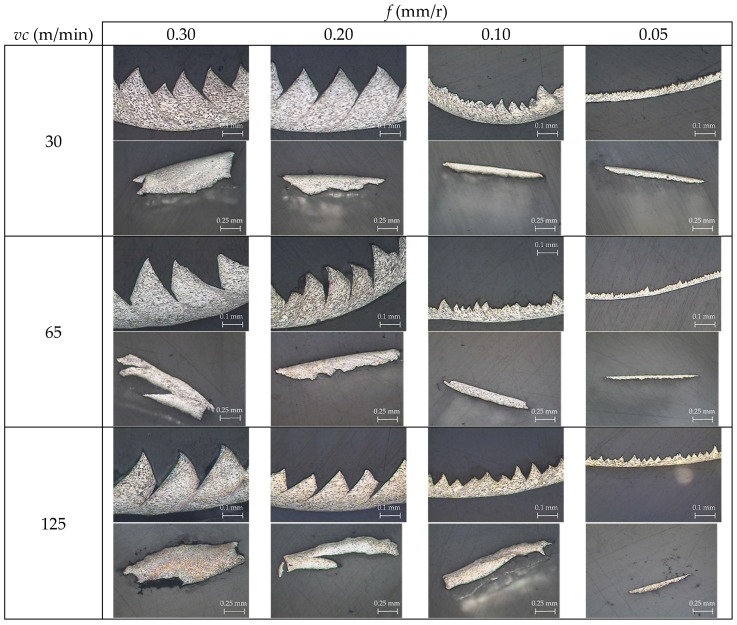
SOM images of the longitudinal (20×, 0.1 mm of each segment) and transverse (10×, 0.25 mm of each segment) chip sections.

Figure 6 shows the SOM images of the longitudinal and transverse chip sections as a function of *v_c_* and *f*. From a qualitative point of view, a higher segmentation level was observed in the longitudinal section when *v_c_* was increased. This increment was enhanced by the feed-rate action. A higher *v_c_* results in an increase in cutting temperature; thus, the aforementioned thermal-softening effect is increased, and the chip is easier to deform. An increase in feed rate implies higher removal rates and cutting forces. As a consequence, the effect of cutting speed is maximized [33]. Therefore, this fact was less noticeable in the low range of feed rates used (0.05–0.10 mm/r). These observations were later quantified using measurements of the chip segment ratio (*G_s_*). Regarding the transverse chip section, no significant changes were observed in the chip width (*b*) as a function of *v_c_* or *f.* Obviously, the transverse section’s area (*A*) increased proportionally to the feed rate.

**Figure 7:**
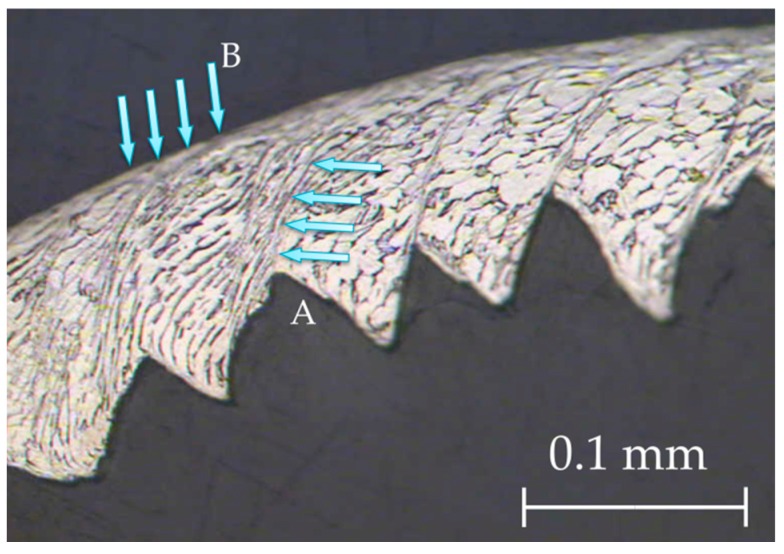
Grain deformation within the chip microstructure (*v_c_* = 125 m/min, *f* = 0.10 mm/r).

When the chip microstructure (Figure 7) was compared with the stock microstructure before machining (Figure 1), grain deformation can be observed in the whole section [4,9]. In addition, two different grain alignments can be noted.

**Figure 8:**
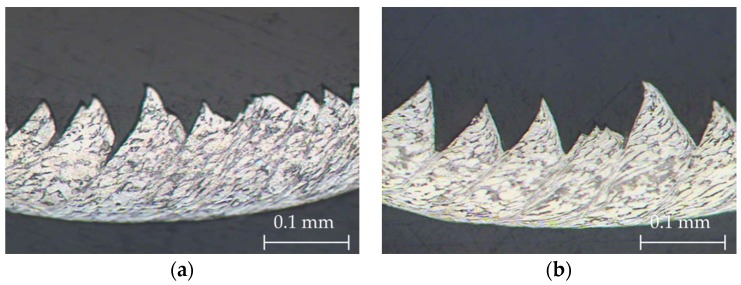
Chip microstructure (40×) for *f* = 0.10 mm/r: (**a**) *v_c_* = 30 m/min, and (**b**) *v_c_* = 125 m/min.

**Figure 9:**
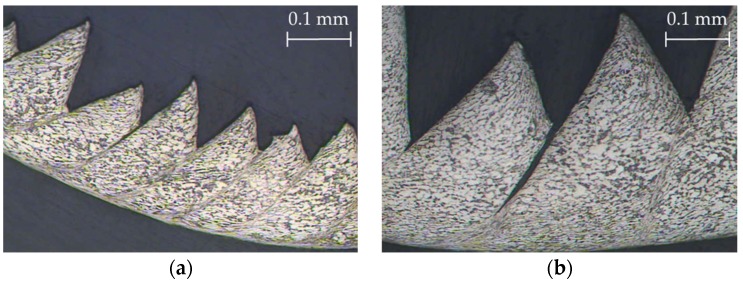
Chip microstructure (20×) at *v_c_* = 125 m/min: (**a**) *f* = 0.20 mm/r, and (**b**) *f* = 0.30 mm/r.

Firstly, there was an alignment in the direction of the adiabatic shear band (primary deformation area, A in Figure 7), due to an intense plastic deformation in this narrow zone [4,45]. This fact was more noticeable when *v_c_* increased, and a more refined grain structure was obtained (Figure 8). The influence of feed rate was more apparent at higher values of cutting speed. The phenomenon of crack initiation and propagation could be observed for high feed rates (0.20–0.30 mm/r), even when high values of cutting speed were applied (Figure 9).

Secondly, an alignment of the grain structure with the main cutting direction was observed, along a very thin layer on the contact surface between the chip and the face of the tool rake (B in Figure 7). This layer, the so-called white layer, was also present in the sub-machined surface of the part, and gave rise to a strain hardening of the machined surface, potentially reducing the alloy’s machinability [2]. This effect was more intense when *v_c_* and *f* were increased.

### 3.2. Geometric Parameters

**Figure 10:**
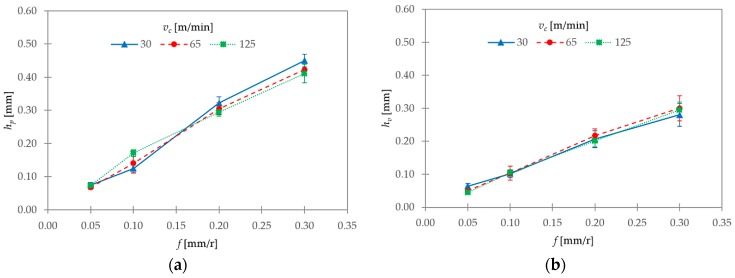
(**a**) Heights of peaks (*h_p_*) and (**b**) valleys (*h_v_*) as a function of *v_c_* and *f*.

**Figure 11:**
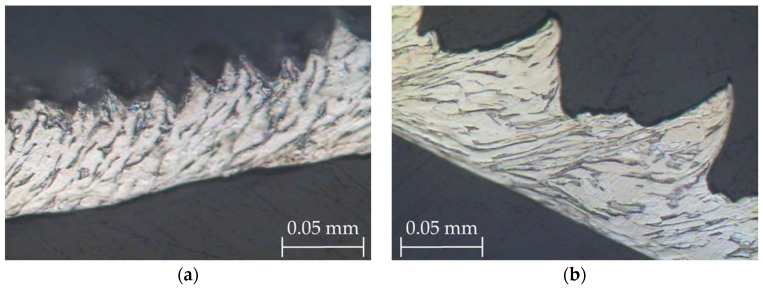
Chip SOM images (100×) for *f* = 0.05 mm/r: (**a**) *v_c_* = 30 m/min, and (**b**) *v_c_* = 125 m/min.

Figure 10 shows the experimental mean values obtained for *h_p_* and *h_v_* as a function of the cutting parameters, *v_c_* and *f*. An almost linear increase with feed rate could be observed for both parameters. This general trend was normal due to the direct proportionality between chip thickness and feed rate. Regarding *v_c_*, a higher influence on *h_p_* was shown for the lowest range of *f* considered. Specifically, for *f* = 0.10 mm/r, *h_p_* showed an increasing trend with *v_c_* (Figure 11). However, for the higher range of *f* (0.20–0.30 mm/r), the highest values of *h_p_* were observed at *v_c_* = 30 m/min. Moreover, no significant changes in *h_v_* were noted as a function of *v_c_*, except for *f* = 0.05 mm/r, where slightly higher values were observed when *v_c_* decreased. An opposite trend was obtained for *f* = 0.30 mm/r.

**Figure 12:**
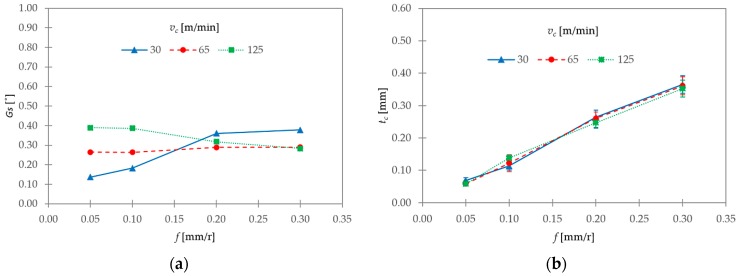
(**a**) Segment ratio (*G_s_*) and (**b**) equivalent chip thickness (*t_c_*) as a function of *v_c_* and *f*.

These observations were complemented by the calculations of the segment ratio (*G_s_*, Equation (3)) and the equivalent chip thickness (*t_c_*, Equation (4)). Their evolutions with *v_c_* and *f* are shown in Figure 12.

Obviously, *t_c_* showed the same trend as that observed for *h_p_* (Figure 12b) as a consequence of the fact that *h_v_* tended to remain more or less constant with cutting parameters. With regards to *G_s_*, the influence of cutting speed was higher for the lowest range of feed rate considered, 0.05–0.10 mm/r (Figure 12a). In this range, the segment ratio tended to increase with *v_c_*. Notwithstanding, *G_s_* tended to remain constant with *f*, except at *v_c_* = 30 m/min, where *G_s_* increased when *f* varied from 0.05 to 0.10 mm/r. A different trend was observed for the highest range of *f* analyzed (0.20–0.30 mm/r). In this case, *G_s_* tended to remain approximately constant with *f*. In addition, the influence of *v_c_* was less noticeable at 65 and 125 m/min, and the highest *G_s_* values corresponded to *v_c_* = 30 m/min. In fact, *G_s_* strongly increased at *v_c_* = 30 m/min, and decreased at *v_c_* = 125 m/min when *f* varied from 0.10 to 0.20 mm/r.

These observations may indicate that *v_c_* was the most influential parameter when a low *f* was used. As a result, thermal softening was the segmentation mechanism that prevailed within this range. By contrast, *f* became more relevant at higher values (0.20–0.30 mm/r). As a result, the mechanism of crack initiation and propagation was more noticeable. Therefore, this mechanism was enhanced by the combination of a low cutting speed and a high feed rate. These observations are in good agreement with previous works [3,4,8,10,33].

**Figure 13:**
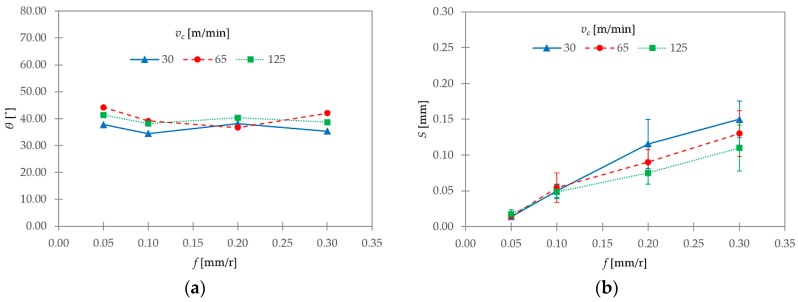
(**a**) Shear angle (*θ*) and (**b**) segment width (*S*) as a function of *v_c_* and *f*.

Figure 13 shows the evolutions of the shear angle (*θ*) and the segment width (*S*) with cutting parameters. The shear angle oscillated from 35° to 44°; as such, it complied with Stabler’s theory for orthogonal cutting [46]. Notwithstanding, no clear trends with *v_c_* and *f* could be observed in the analyzed interval. Some authors found a general trend of increasing *θ* for low ranges of cutting speed (0–40 m/min). However, scattered results were found between 40 and 140 m/min [33], in good agreement with the results exposed in this work. Thus, these results are within the normal variation of the measurement process.

Regarding segment width (*S*), this parameter showed a general trend of increase with *f*. On the other hand, *S* decreased with *v_c_*. This trend was more noticeable for the highest values of *f* used (0.20–0.30 mm/r). This fact can be explained taking into account that an increase in cutting speed results in an increase in cutting temperature. Because of the low thermal conductivity of the Ti6Al4V alloy, the primary shear zone becomes more adiabatic, and the appearance of adiabatic shear bands is favored. Thus, the segmentation frequency is increased [4,9,33].

**Figure 14:**
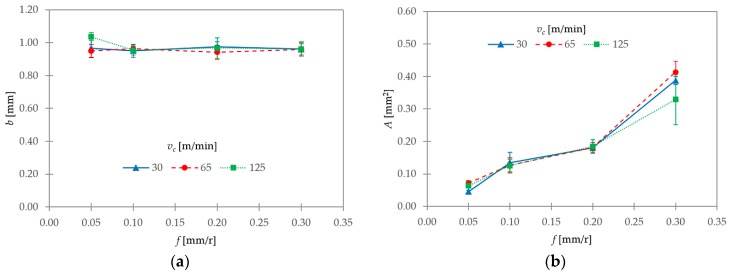
(**a**) Chip width (*b_v_*) and (**b**) chip transverse area (*A*) as a function of *v_c_* and *f*.

Figure 14 shows two different geometric parameters of the transverse chip section and their evolutions with *v_c_* and *f*: the chip width (*b*; Figure 14a) and the chip transverse area (*A*; (Figure 14b). A general trend of remaining constant was observed for *b*, regardless of changes in *v_c_* and *f*. Its value was very close to the theoretical value (*a_p_* = 1 mm). As a result, the hypothesis of constant volume and plain strain during machining could be assumed.

As seen in Figure 14b, the chip transverse area (*A*) tended to increase with *f*, and it was independent of *v_c_*. Its value was measured along the adiabatic shear band. Therefore, *A* was very close to the product of *h_v_* and *b.* Its value only showed more dispersion for *f* = 0.30 mm/r. A possible explanation is that the transverse area was less homogenous for 0.30 mm/r, and, as a result, more difficult to measure (Figure 6).

**Figure 15:**
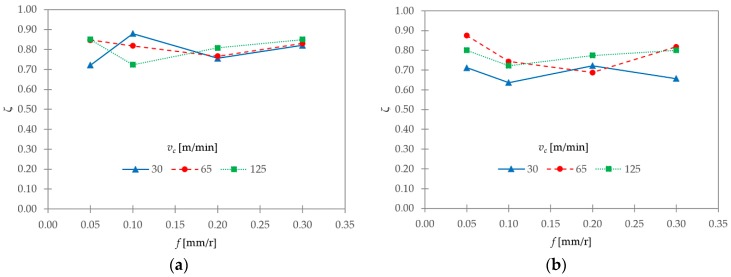
Shrinkage factor (*ζ*) as a function of *v_c_* and *f*, calculated via (**a**) Equation (5), and (**b**) via Equation (2).

The shrinkage factor (*ζ*) provides important information about the chip’s total strain, the chip’s springback, and the consumed energy in the chip’s plastic deformation, among others [20,42]. The hypothesis of plain strain and constant volume allows calculating the shrinkage factor (*ζ*) via Equation (2), where *γ* = 7° and *θ* is the experimentally obtained shear angle. In addition, *ζ* can be calculated with Equation (5), using the theoretical chip thickness before machining (*t*_0_) and the equivalent chip thickness (*t_c_*). For the selected machining configuration (*κ_r_* = 90°, main cutting edge angle), *t*_0_ was equal to *f*. Figure 15a,b show the evolution of ζ as a function of *v_c_* and *f*, calculated with Equations (2) and (5), respectively.

(5) $$\;\zeta =\frac{t_{0}}{t_{c}}\;$$

In the first case (Figure 15a) *ζ* varied from 0.72 to 0.88. In the second case (Figure 15b) *ζ* varied from 0.64 to 0.87. In both cases, there was no a clear trend of increase or decrease with *v_c_* or *f*. In general, the lowest values were obtained at *v_c_* = 30 m/min, although some singularities could be observed for *f* = 0.10 mm/r (Figure 15a). Thus, low cutting speeds resulted in higher deformation rates.

### 3.3. Parametric Relantioships

As mentioned in the introduction, the prediction of chip morphology before machining is of crucial importance [2,8]. In this research, various parametric relationships were developed from the experimental data. These relationships allow predicting the evolution of chip-geometric parameters as a function of the cutting parameters analyzed, *v_c_* and *f*. To establish a global model was not intended, due to the large number of variables which influence the process, in addition to the cutting parameters. However, obtaining simpler models for a direct industrial application was interesting [42].

These relationships were obtained for the parameters with a strong dependence on cutting parameters (*h_p_*, *h_v_*, *t_c_*, *G_s_*, *S*, and *A*), and for those that showed a weaker dependence (*θ* and *ζ*). Obviously, no relationship was obtained for *b*, given its strong trend of remaining practically constant.

To achieve this objective, various mathematical models were tested. Among them, a potential model, as shown in Equation (6), exhibited the best fit to the experimental data for all geometric parameters (*GP*) studied.

(6) $$GP=K\cdot v_{c}^{x}\cdot f^{y}$$

In Equation (6), K, x, and y are constants. Table 3 provides the results for the constants for each geometric parameter (*GP*) after fitting this model to the experimental data. An additional column was added, with the adjusted coefficient of determination (R^2^).

As can be seen in Table 3, *h_p_*, *h_v_*, *t_c_*, *S*, and *A* showed a good fit to the proposed model (R^2^ values between 0.92 and 0.98). The model coefficients for these geometric parameters indicated a strong influence of *f*, with the y coefficient almost linear. Because of the lower value of x, the influence of *v_c_* was almost negligible. This fact is in good agreement with the experimental observations.

**Figure 16:**
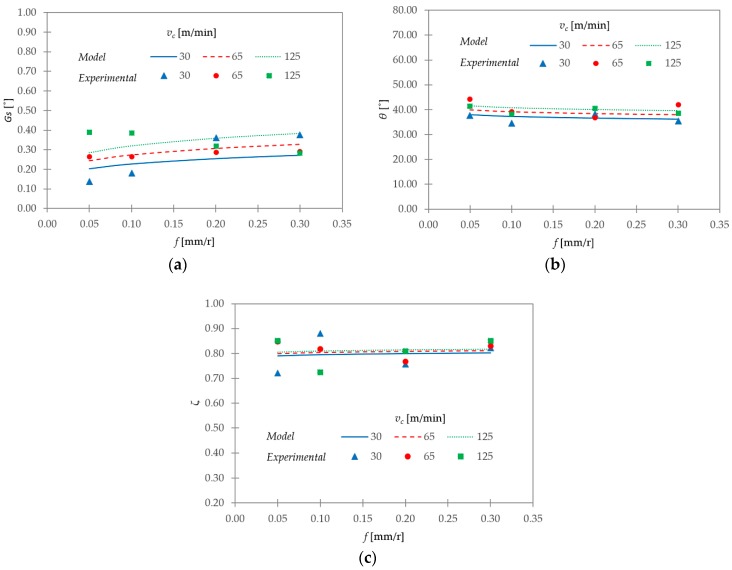
Comparisons between the experimental data and the proposed model for (**a**) *G_s_*, (**b**) *θ*, and (**c**) *ζ.*

With regards to *G_s_*, *θ*, and *ζ*, the model exhibited a lower fit (R^2^ values between 0.52 and 0.67). Figure 16 contrasts the experimental data and the proposed model for these parameters.

As observed in Figure 16a, the *G_s_* model showed a good fit at *v_c_* = 65 m/min, regardless of changes in *f*. However, this model underestimated *G_s_* at *v_c_* = 30 m/min, and overestimated it at *v_c_* = 125 m/min in the lower range of *f* studied (0.05–0.10 mm/r). An opposite trend was observed for higher *f* (0.10–0.20 mm/r). Regarding *θ* (Figure 16b) the model showed, in general, a good adjustment at *v_c_* = 30 and 125 m/min. At *v_c_* = 65 m/min, it seemed to overestimate it for *f* = 0.05 and 0.30 mm/r. Finally, because there was no clear tendency of *ζ* with cutting parameters, the model was only useful to show an average trend (very close to *ζ* = 0.8, and almost equal to K = 0.77 in the model) within the studied *v_c_* and *f* intervals (Figure 16c).

## 4. Conclusions

In this work, an experimental analysis of the influence of cutting speed and feed rate on chip morphology and geometry was carried out, during the dry machining of a Ti6Al4V alloy.

In general, the chip morphology was segmented, and it remained continuous across wide ranges of *v_c_* and *f*. This was a consequence of the high plasticity levels of this alloy and its low thermal conductivity. This fact became more noticeable when *v_c_* was increased and *f* was reduced. From a qualitative point of view, a higher segmentation level was observed in the chip’s longitudinal section when *v_c_* was increased. This increase was enhanced by *f*.

Regarding chip microstructure, a grain deformation was observed in the whole section. In addition, two different grain alignments were noticed within two different areas: the adiabatic shear band and the contact surface between the chip and the face of the tool rake. Both alignments were more noticeable when *v_c_* was increased.

Various chip-geometric parameters (longitudinal and transverse chip sections) were measured, and their evolutions with *v_c_* and *f* were studied.

An almost linear increase with *f* was observed in the height of peaks (*h_p_*), the height of valleys (*h_v_*), the equivalent chip thickness (*t_c_*), and the area on the transverse section (*A*). This general trend was expected, due to the direct proportionality between chip thickness and *f*. Regarding *v_c_*, a higher influence on *h_p_* was observed in the lowest range of *f* considered. No significant changes in *h_v_* were noted as a function of *v_c_*. The equivalent chip thickness (*t_c_*) showed a similar trend to that observed for *h_p_*, because *h_v_* tended to remain more or less constant with cutting parameters.

The segment ratio (*G_s_*) showed a general trend of increasing with *v_c_*. This trend was stronger for the lowest range of *f*. The feed rate showed a lesser influence on this parameter. The segment width (*S*) exhibited a general trend of increasing with *f*, and of decreasing with *v_c_*. This trend was more noticeable for the highest values of *f*. The shear angle (*θ*) oscillated from 35° to 44°. Notwithstanding, no clear trends with *v_c_* and *f* were found. A general trend of remaining constant was observed for chip width (*b*), regardless of changes in *v_c_* and *f*. As a result, the hypothesis of constant volume and plain strain during machining could be assumed. The shrinkage factor (ζ) varied from 0.64 to 0.88. No clear trends for this parameter were found as a function of *v_c_* or *f*. Notwithstanding, the lowest values were obtained, in general, at the lowest value of *v_c_*. Thus, low cutting speeds resulted in higher deformation rates.

Various parametric relationships were developed from the experimental data. These relationships allowed predicting the evolution of the chip-geometric parameters as a function of the cutting parameters. Several mathematical models were tested, and the potential model exhibited the best fit. The geometric parameters, *h_p_*, *h_v_*, *t_c_*, *S*, and *A*, showed a good fit to the proposed model. This model exhibited a weaker fit for *G_s_*, *θ*, and *ζ*.

It is necessary to point out that all these observations are only valid within the tested range of cutting parameters.

## Figures and Tables

**Table 1:** Composition of the machined alloy (wt.%).

Alloy	C	Fe	N	O	Al	V	Ti
Part	0.08	0.164	0.05	0.05	5.47	4.09	Balance

**Table 2:** Cutting parameters.

Feed Rate, *f* (mm/r)	Cutting Speed, *v_c_* (m/min)	Cutting Depth, *a_p_* (mm)
0.05	30 65 125	1
0.10		
0.20		
0.30		

**Table 3:** Model coefficients.

Geometric Parameter (*GP*)	K	x	y	R^2^
Height of peak (*h_p_*)	1.38	0.02	1.01	0.98
Height of valley (*h_v_*)	1.20	−0.05	0.98	0.97
Chip thickness (*t_c_*)	1.29	−0.01	0.99	0.98
Segment ratio (*G_s_*)	0.15	0.24	0.17	0.52
Segment width (*S*)	0.87	−0.10	1.16	0.94
Transverse area (*A*)	0.94	0.02	0.95	0.92
Shear angle (*θ*)	28.43	0.06	−0.03	0.67
Shrinkage factor (*ζ*)	0.77	0.15	0.02	0.61
